# Crystal structure of bis­[(*S*)-2-(2-hy­droxy­benzyl­amino)-4-methyl­penta­noato-κ^2^ *N*,*O* ^1^](1,10-phenanthroline-κ^2^ *N*,*N*′)cadmium dihydrate

**DOI:** 10.1107/S2056989018013877

**Published:** 2018-10-12

**Authors:** Md. Serajul Haque Faizi, Necmi Dege, James Pogrebetsky, Turganbay S. Iskenderov

**Affiliations:** a Department of Chemistry, Langat Singh College, Babasaheb Bhimrao Ambedkar Bihar, University, Muzaffarpur, Bihar, India; b Ondokuz Mayis University, Arts and Sciences Faculty, Department of Physics, 55139 Samsun, Turkey; c Department of Chemistry, Taras Shevchenko National University of Kyiv, 64, Volodymyrska Str., 01601 Kiev, Ukraine

**Keywords:** crystal structure, Cd^II^ complex, distorted octa­hedral coordination, O— H⋯O hydrogen bonding, π—π stacking inter­actions., crystal structure

## Abstract

The asymmetric unit of the mononuclear mixed-ligand title complex, [Cd(C_13_H_18_NO_3_)_2_(C_12_H_8_N_2_)]·2H_2_O, contains two crystallographically independent mol­ecules that differ insignificantly in their geometrical parameters. In both, the Cd^II^ cation lies on a twofold rotation axis and is coordinated in a distorted octa­hedral fashion to two monodeprotonated residues of the l-leucine-derived ligand (*S*)-2-(2-hy­droxy­benzyl­amino)-4-methyl­penta­noic acid (**L**), as well as to a 1,10-phenanthroline ligand in a κ^2^ *N*,*N*′ mode. The former coordinate in an *N*,*O*-chelating mode, exhibiting a *trans*-*N*,*N*′ mutual disposition. The phenolic oxygen donor groups remain protonated and do not coordinate to the cation but take part in intra- and inter­molecular hydrogen bonds. In the crystal, O—H⋯O hydrogen bonding results in the formation of a three-dimensional network structure. The contribution to the electron density of two disordered water mol­ecules was removed with the SQUEEZE procedure in *PLATON* [Spek (2015 ▸). *Acta Cryst.* C**71**, 9–18]. The studied crystal was refined as a two-component inversion twin. The title complex was also characterized by IR and ^1^H NMR spectroscopic methods.

## Abstract 2

The crystal structure of [Cd(C_13_H_18_NO_3_)_2_(C_12_H_8_N_2_)] is non-centrosymmetric. Two complex mol­ecules with similar bond lengths and angles are present in the asymmetric unit, each exhibiting a distorted octa­hedral N_4_O_2_ coordination environment around the Cd^II^ ion.

## Chemical context   

Schiff base metal complexes are an important research area with respect to inorganic and supra­molecular chemistry (Burkhardt *et al.*, 2008 ▸; Przybylski *et al.*, 2009 ▸; Moroz *et al.*, 2012 ▸). Such compounds have been found to exhibit a number of properties among which are anti­bacterial, anti­fungal, anti­tumor, herbicidal activities (Asadi *et al.*, 2011 ▸), as well as having applications in pharmaceutical, agricultural and industrial chemistry (Anis *et al.*, 2013 ▸). Unlike oximes, another azomethine ligand family (Sliva *et al.*, 1997 ▸; Penkova *et al.*, 2010 ▸; Pavlishchuk *et al.*, 2010 ▸), Schiff base ligands containing additional polar or acidic groups are known for their enhanced reactivity and, as a consequence, instability upon coordination to metals (Casella & Gullotti, 1983 ▸). Thus, attempts to isolate Schiff bases derived from amino­hydroxamic acids resulted in cyclization under the formation of 2-substituted 3-hy­droxy­imidazolidine-4-ones (Iskenderov *et al.*, 2009 ▸). In attempts to achieve stable polydentate ligand systems retaining the initial donor sets, it was found that reduction of Schiff bases to amines allows the formation of stable complexes (Koh *et al.*, 1996 ▸). Phenanthroline and phenanthroline-derived ligands also have important roles in many fields (Faizi & Sharkina, 2015 ▸; Faizi *et al.*, 2017 ▸). Herein we report the synthesis and structure of a new hydrated cadmium complex, [Cd(C_13_H_18_NO_3_)_2_(C_12_H_8_N_2_)]·2H_2_O, with a phenanthroline ligand and two ligands derived from l-leucine.
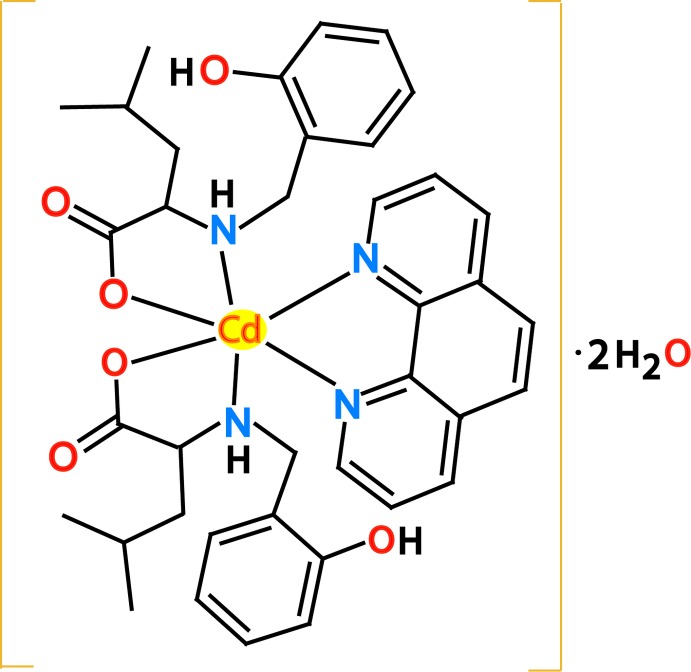


## Structural commentary   

**Figure 1:**
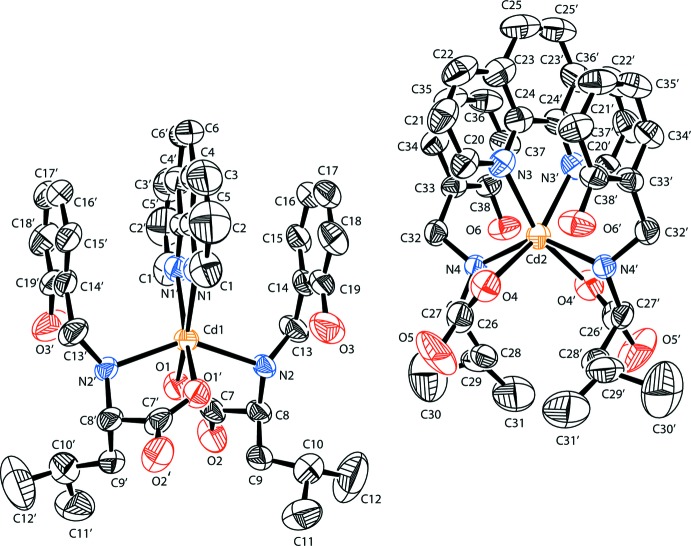
Structures of the two complex mol­ecules in the title compound. Displacement ellipsoids are drawn at the 40% probability level. Atoms with primed labels are generated by the symmetry operations −*x* + 1, *y*, −*z* + 1 for complex Cd1 and −*x* + 1, *y*, −*z* + 2 for complex Cd2.

The asymmetric unit of the title complex contains two mononuclear mol­ecules (Fig. 1 ▸). In each, the metal cation is located on a twofold rotation axis and is coordinated by three chelating ligands, leading to a distorted octa­hedral N_4_O_2_ coordination sphere. The mixed-ligand complex is made up from one neutral phenanthroline ligand and two residues of the monodeprotonated l-leucine-derived ligand **L**. The **L** ligands of each complex are *trans*-*N*,*N*′ disposed with respect to each other and comprise the chiral atoms C8 for the first and C27 for the second mol­ecule. The Cd—O and Cd—N bond lengths in the first mol­ecule are virtually the same in the Cd1O_4_N_2_ octa­hedron with Cd1—O1, Cd1—N1 and Cd1—N2 = 2.346 (3), 2.341 (4) and 2.315 (4) Å, respectively. The second mol­ecule also exhibits similar geometrical parameters [Cd2—O4, Cd2—N3 and Cd2—N4 = 2.322 (4), 2.351 (5) and 2.339 (4) Å, respectively]. All three sets of ligands form five-membered chelate rings. Unlike the essentially planar chelate rings formed by the phenanthroline ligands, the ones involving the l-leucine-derived ligands exhibit a λ-conformation in both complex mol­ecules. The deviations of the carbon atoms from the planes defined by the central atom and donor atoms are 0.258 (6) Å for C7, 0.599 (7) Å for C8, −0.417 (7) Å for C26 and 0.632 (5) Å for C27. In the second mol­ecule, the highest deviations are found to be 0.160 and 0.232 Å for O4 and N4, respectively. The N—Cd—O and N—Cd—N bite angles are 73.01 (13) and 71.2 (2)°, respectively, for the first mol­ecule and 72.40 (14) and 70.8 (2)° for the second. The phenolic O—H group remains protonated and is non-coordinating, albeit participating in an extensive inter­molecular hydrogen-bonding network. Intra­molecular hydrogen bonds are also found to exist and take place between atoms H2*A* and O3 as well as between H4 and O6 of the l-leucine-derived ligands. To a minor extent, intra­molecular C—H⋯O inter­actions are also present between a methyl­ene group and O4 (Table 1 ▸).

## Supra­molecular features   

**Figure 2:**
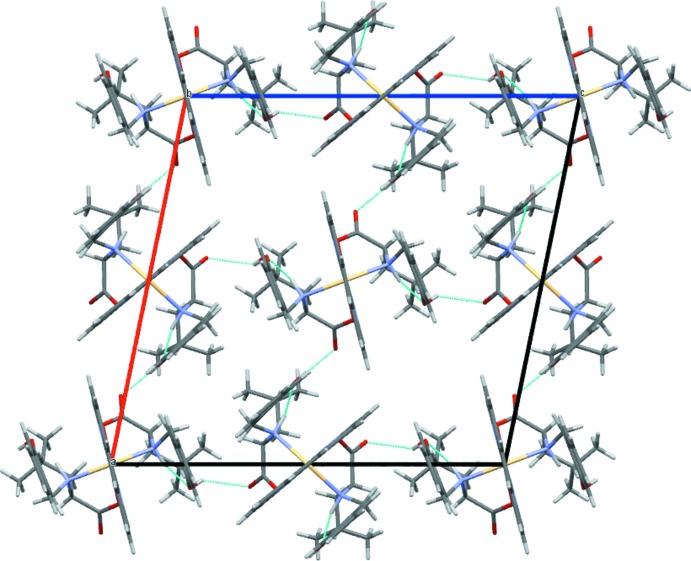
The crystal packing of the title compound viewed along [010]. Hydrogen bonds are shown as dashed lines (see Table 1 ▸ for numerical details).

In the crystal structure, the complex mol­ecules are linked *via* hydrogen-bonding inter­actions between phenolic O—H and C—O groups of l-leucine-derived ligands (Table 1 ▸, Fig. 2 ▸). π–π inter­actions take place between the central phenanthroline ring and the C14–C19 rings of two leucine-derived **L** ligands with distances between the centroids of the aromatic fragments being 3.813 (4) Å for the first mol­ecule. The stacking inter­actions of the second mol­ecule are between C33–C38 rings of two l-leucine-derived ligands **L** and the C23–C25/C23′–C25′(–*x* + 1, *y*, –*z* + 2) phenanthroline fragment with a centroid-to-centroid distance of 3.773 (4) Å.

## Database survey   

A search in the Cambridge Structural Database (Version 5.39, last update February 2018; Groom *et al.*, 2016 ▸) revealed only one precedent of a Cd^II^ complex with a 2-hy­droxy­benzyl derivative of an amino acid (refcode WARLIL). In this mononuclear complex, the phenolic and *β*-carb­oxy­lic groups are deprotonated. The *N*-(2-hy­droxy­benz­yl)-d,l-aspartic acid residue coordinates in an (*O*,*N*,*O*′)-tridentate mode including the phenolic O atom (Lou *et al.*, 2005 ▸). This differs from the title compound in which the phenolic group is protonated and is non-coordinating. The second O atom of the *β*-carb­oxy­lic group bridges the neighbouring Cd^II^ units into a polymeric chain. In addition, there are four structures of complexes of homologous zinc and with 2-hy­droxy­benzyl derivatives of alanine (refcodes AZIROQ, AZIRUW, NOLYIW, NOLYOC). These compounds have a Zn_2_O_2_ binuclear core, and the ligands also coordinate in an (*O*,*N*,*O*′)-tridentate manner, with an additional *μ*_2_-mode for the phenolic O atom (Lou *et al.*, 2004 ▸; Ranford *et al.*, 1998 ▸).

## Synthesis and crystallization   

**Synthesis of (** ***S*** **)-2-(2-hy­droxy­benzyl­amino)-4-methyl­penta­noic acid (L)**

A mixture of l-leucine (1.00 g, 7.62 mmol) and LiOH·H_2_O (0.323 g, 7.62 mmol) in methanol (25 ml) was stirred for 10 min to dissolve. A methano­lic solution of *o*-salicyl­aldehyde (0.930 g, 7.62 mmol) was added dropwise to the above solution whereby the colour of the solution turned to yellow. Stirring was continued for 30 min before the solution was treated with NaBH_4_ (0.580 g, 15.3 mmol), leading to a colourless solution. The solvent was evaporated under reduced pressure, and the resulting solid was dissolved in water. The clear solution was then acidified with diluted HCl (pH ∼5–7). The ligand precipitated as a white solid. The suspension was filtered, and the residue was washed thoroughly with water. The solid was dried in a vacuum desiccator (yield 1.65 g, 88%). Because of its poor solubility, the ^1^H NMR spectrum for the ligand was recorded as the lithium salt of the ligand, prepared by adding 2 equiv. of LiOH·3H_2_O in CD_3_OD. ^1^H NMR Li_2_ **L** (CD_3_OD, 400 MHz, ppm): 0.76 (*d*, 3H, H^j^) , 0.81 (*d*, 3H, H^i^) , 1.36 (*m*, 1H, H^g^) , 1.41 (*m*, 1H, H^g′^), 1.67 (*m*, 1H, H^h^) , 3.07 (*dd*, 1H, H^f^) , 3.65 (*d*, 1H, H^e^) , 3.94 (*d*, 1H, H^e′^), 6.35 (*t*, 1H, H^c^) , 6.45 (*d*, 1H, H^a^) , 6.94 (*m*, 2H, H^b,d^). *m*/*z* (ESI–MS, [Li**L**]^−^); calculated: 242.22, found 242.02. IR (KBr, cm^−1^) ν(COO)_asym_ 1600 (*s*), 1593 (*s*); ν (COO)_sym_ 1393 (*m*), cm^−1^.

**Synthesis of [Cd(L)_2_(phen)]·(H_2_O)_2_]**

A methano­lic solution of Cd(NO_3_)_2_·4H_2_O (0.130 g, 0.421 mmol) was added under stirring to 20 ml of a methano­lic solution of **L** (0.200 g, 0.843mmol) and NaOH (0.034 g, 0.843 mmol), followed by addition of phenanthroline monohydrate (0.076 g, 0.421mmol) in 5 ml of methanol. A clear solution was formed within half an hour under constant stirring. After 2 h, the solvent was evaporated to dryness. The residue was subsequently washed with methanol and diethyl ether, and finally dried under vacuum. Empirical formula [Cd(**L**)_2_(phen)]·2H_2_O. Yield: 60%. [Cd(**L**)_2_(phen)]·2H_2_O: IR (KBr, cm^−1^) ν(COO)_asym_ 1594, ν(COO)_sym_ 1384, ν(phenolic, CO) 1257. ^1^H NMR [Cd(**L**)_2_(phen)]·2H_2_O] (DMSO, 400 MHz. ppm): 0.6 (*s*, *broad*, 3H^J^), 0.7 (*s*, *broad*, 3H^i^), 1.3 (*s*, *broad*, 1H^g^), 1.5 (*s*, *broad*, H^g′^), 2.7 (*s*, *broad*, 1H^f^), 2.9 (*s*, *broad*, 2H^e,e′^), 6.6 (*s*, *broad*, 1H^d^), 6.4 (*s*, *broad*, 1H^c^), 6.6 (*s*, *broad*, 1H^b^), 6.1 (*s*, *broad*,1H^a^), 8.0 (*s*, *broad*, 2H^n^), 8.1 (*s*, *broad*, 2H^m^), 8.7 (*s*, *broad*, 2H^l^), 9.1 (*s*, *broad*, 2H^k^). ESI–Mass (-ve) at 829.18 (calculated 829.18). Suitable needle-shaped crystals for X-ray data collection were obtained by slow evaporation of a methanol: DMF (2:1 *v*:*v*) solution within a week.

## Refinement   

Crystal data, data collection and structure refinement details are summarized in Table 2 ▸. Atoms O3, C3, C4 and C6 showed highly anisotropic displacement parameters and were modelled using the ISOR instruction in *SHELXL* (Sheldrick, 2015 ▸). The H atoms of the phenolic OH group were located from a difference-Fourier map and were constrained to ride on their parent atoms, with O—H = 0.82 Å and with *U*_iso_(H) = 1.5*U*_eq_(O). All C-bound H atoms were positioned geom­etrically and refined using a riding model with C—H = 0.93 Å and with *U*_iso_(H) = 1.2*U*_eq_(C).

After unsuccessful attempts to model disordered solvent mol­ecules, their contributions to the diffraction data were removed by using the SQUEEZE routine in *PLATON* (Spek, 2015 ▸). *PLATON* calculated a solvent-accessible void volume in the unit cell of 629 Å^3^ (15.4% of the total cell volume), corresponding to 151 electrons (residual electron density after the last refinement cycle) per unit cell, or 37.75 electrons per one complex mol­ecule. This number agrees with two water mol­ecules. Although not modelled in the refined structure, the two water mol­ecules are included in the formula and other crystallographic data.

## Supplementary Material

Crystal structure: contains datablock(s) I. DOI: 10.1107/S2056989018013877/wm5464sup1.cif

Structure factors: contains datablock(s) I. DOI: 10.1107/S2056989018013877/wm5464Isup2.hkl

CCDC reference: 1534691

Additional supporting information:  crystallographic information; 3D view; checkCIF report

## Figures and Tables

**Table 1:** Hydrogen-bond geometry (Å, °)

*D*—H⋯*A*	*D*—H	H⋯*A*	*D*⋯*A*	*D*—H⋯*A*
O6—H6*A*⋯O2^i^	0.82	1.83	2.645 (5)	174
N4—H4⋯O6	0.98	2.07	2.763 (5)	126
N2—H2*A*⋯O3	0.98	2.09	2.795 (6)	127
O3—H3*A*⋯O5	0.82	2.33	2.951 (9)	133
C28—H28*A*⋯O4^ii^	0.97	2.67	3.470 (7)	141

Symmetry codes: (i) 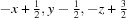; (ii) 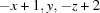.

**Table 2:** Experimental details

Crystal data	
Chemical formula	[Cd(C_13_H_18_NO_3_)_2_(C_12_H_8_N_2_)]·2H_2_O
*M*_r_	801.22
Crystal system, space group	Monoclinic, *I*2
Temperature (K)	293
*a*, *b*, *c* (Å)	18.0171 (6), 12.2561 (3), 18.8597 (9)
β (°)	101.582 (3)
*V* (Å^3^)	4079.8 (3)
*Z*	4
Radiation type	Mo *K*α
μ (mm^−1^)	0.59
Crystal size (mm)	0.19 × 0.12 × 0.09

Data collection	
Diffractometer	Bruker SMART CCD
Absorption correction	Multi-scan (*SADABS*; Bruker, 2011 ▸)
*T*_min_, *T*_max_	0.867, 0.942
No. of measured, independent and observed [*I* > 2σ(*I*)] reflections	22798, 7944, 6342
*R*_int_	0.037
(sin θ/λ)_max_ (Å^−1^)	0.617

Refinement	
*R*[*F* ^2^ > 2σ(*F* ^2^)], *wR*(*F* ^2^), *S*	0.034, 0.076, 0.99
No. of reflections	7944
No. of parameters	444
No. of restraints	37
H-atom treatment	H-atom parameters constrained
Δρ_max_, Δρ_min_ (e Å^−3^)	0.22, −0.18
Absolute structure	Refined as an inversion twin
Absolute structure parameter	−0.03 (3)

Computer programs: *SMART* and *SAINT* (Bruker, 2011 ▸), *SHELXTL* (Sheldrick, 2008 ▸), *SHELXL2018* (Sheldrick, 2015 ▸), *ORTEP-3 for Windows* and *WinGX* (Farrugia, 2012 ▸) and *Mercury* (Macrae *et al.*, 2008 ▸).

## supplementary crystallographic information

### Crystal data

**Table:**

[Cd(C_13_H_18_NO_3_)_2_(C_12_H_8_N_2_)]·2H_2_O	*F*(000) = 1584
*M**_r_* = 801.22	*D*_x_ = 1.304 Mg m^−^^3^
Monoclinic, *I*2	Mo *K*α radiation, λ = 0.71073 Å
*a* = 18.0171 (6) Å	Cell parameters from 2245 reflections
*b* = 12.2561 (3) Å	θ = 1.8–26.0°
*c* = 18.8597 (9) Å	µ = 0.59 mm^−^^1^
β = 101.582 (3)°	*T* = 293 K
*V* = 4079.8 (3) Å^3^	Needle, colorless
*Z* = 4	0.19 × 0.12 × 0.09 mm

### Data collection

**Table:**

Bruker SMART CCD diffractometer	7944 independent reflections
Radiation source: fine-focus sealed tube, x-ray	6342 reflections with *I* > 2σ(*I*)
Graphite monochromator	*R*_int_ = 0.037
phi and ω scans	θ_max_ = 26.0°, θ_min_ = 1.8°
Absorption correction: multi-scan (SADABS; Bruker, 2011)	*h* = −22→21
*T*_min_ = 0.867, *T*_max_ = 0.942	*k* = −15→15
22798 measured reflections	*l* = −23→23

### Refinement

**Table:**

Refinement on *F*^2^	Hydrogen site location: inferred from neighbouring sites
Least-squares matrix: full	H-atom parameters constrained
*R*[*F*^2^ > 2σ(*F*^2^)] = 0.034	*w* = 1/[σ^2^(*F*_o_^2^) + (0.0365*P*)^2^] where *P* = (*F*_o_^2^ + 2*F*_c_^2^)/3
*wR*(*F*^2^) = 0.076	(Δ/σ)_max_ = 0.036
*S* = 0.99	Δρ_max_ = 0.22 e Å^−^^3^
7944 reflections	Δρ_min_ = −0.18 e Å^−^^3^
444 parameters	Absolute structure: Refined as an inversion twin
37 restraints	Absolute structure parameter: −0.03 (3)

### Special details

**Table:**

Geometry. All esds (except the esd in the dihedral angle between two l.s. planes) are estimated using the full covariance matrix. The cell esds are taken into account individually in the estimation of esds in distances, angles and torsion angles; correlations between esds in cell parameters are only used when they are defined by crystal symmetry. An approximate (isotropic) treatment of cell esds is used for estimating esds involving l.s. planes.
Refinement. Refined as a 2-component inversion twin.

### Fractional atomic coordinates and isotropic or equivalent isotropic displacement parameters (Å^2^)

**Table:**

	*x*	*y*	*z*	*U*_iso_*/*U*_eq_	
Cd1	0.500000	0.69148 (3)	0.500000	0.05478 (18)	
C1	0.3532 (4)	0.5367 (7)	0.4350 (4)	0.094 (2)	
H1	0.328791	0.603070	0.423336	0.113*	
C2	0.3123 (6)	0.4396 (10)	0.4188 (6)	0.136 (4)	
H2	0.261275	0.441306	0.396832	0.163*	
C3	0.3476 (8)	0.3454 (10)	0.4352 (6)	0.142 (4)	
H3	0.320426	0.281053	0.424184	0.171*	
C4	0.4239 (6)	0.3391 (6)	0.4682 (4)	0.106 (2)	
C5	0.4604 (3)	0.4394 (4)	0.4823 (3)	0.0693 (15)	
C6	0.4641 (6)	0.2410 (6)	0.4860 (6)	0.146 (6)	
H6	0.438503	0.174909	0.477383	0.176*	
C7	0.3702 (3)	0.8567 (5)	0.5040 (3)	0.0707 (15)	
C8	0.4129 (4)	0.8566 (6)	0.5827 (4)	0.0677 (19)	
H8	0.376062	0.862966	0.614269	0.081*	
C9	0.4657 (4)	0.9528 (5)	0.5960 (3)	0.0820 (18)	
H9A	0.436890	1.017813	0.578965	0.098*	
H9B	0.503823	0.943726	0.566820	0.098*	
C10	0.5066 (6)	0.9725 (7)	0.6751 (4)	0.121 (3)	
H10	0.533040	0.904880	0.692573	0.145*	
C11	0.5670 (6)	1.0626 (7)	0.6790 (5)	0.152 (4)	
H11A	0.600500	1.044664	0.646987	0.228*	
H11B	0.542692	1.131051	0.664725	0.228*	
H11C	0.595593	1.068085	0.727635	0.228*	
C12	0.4540 (7)	0.9980 (11)	0.7240 (6)	0.208 (6)	
H12A	0.416817	0.941072	0.720849	0.313*	
H12B	0.481962	1.003153	0.772901	0.313*	
H12C	0.429061	1.066120	0.709991	0.313*	
C13	0.4116 (3)	0.6711 (5)	0.6333 (4)	0.0833 (18)	
H13A	0.365937	0.653468	0.598276	0.100*	
H13B	0.396256	0.704215	0.674837	0.100*	
C14	0.4537 (3)	0.5672 (5)	0.6570 (3)	0.0637 (14)	
C15	0.4194 (4)	0.4692 (7)	0.6421 (3)	0.0750 (16)	
H15	0.370191	0.467626	0.614955	0.090*	
C16	0.4535 (5)	0.3729 (6)	0.6649 (4)	0.089 (2)	
H16	0.428382	0.307134	0.653054	0.106*	
C17	0.5248 (5)	0.3745 (6)	0.7052 (4)	0.096 (2)	
H17	0.548436	0.309439	0.722026	0.116*	
C18	0.5618 (4)	0.4705 (7)	0.7211 (4)	0.099 (2)	
H18	0.611238	0.471017	0.747584	0.119*	
C19	0.5264 (4)	0.5667 (5)	0.6981 (3)	0.0790 (17)	
N1	0.4256 (3)	0.5362 (4)	0.4664 (2)	0.0674 (12)	
N2	0.4565 (2)	0.7519 (3)	0.6006 (2)	0.0576 (10)	
H2A	0.501263	0.769513	0.637666	0.069*	
O1	0.3930 (2)	0.7999 (3)	0.4576 (2)	0.0728 (10)	
O2	0.3145 (3)	0.9197 (4)	0.4897 (3)	0.1030 (15)	
O3	0.5641 (3)	0.6627 (4)	0.7132 (3)	0.130 (2)	
H3A	0.542946	0.699788	0.739444	0.194*	
Cd2	0.500000	0.46530 (4)	1.000000	0.05859 (18)	
C20	0.5982 (4)	0.3122 (7)	0.9104 (4)	0.076 (2)	
H20	0.614551	0.379213	0.896042	0.091*	
C21	0.6251 (4)	0.2141 (7)	0.8843 (4)	0.094 (2)	
H21	0.659847	0.216857	0.853995	0.113*	
C22	0.6000 (4)	0.1167 (7)	0.9037 (4)	0.098 (2)	
H22	0.615858	0.052420	0.885145	0.118*	
C23	0.5505 (3)	0.1129 (5)	0.9514 (4)	0.0829 (19)	
C24	0.5265 (3)	0.2132 (4)	0.9755 (3)	0.0661 (14)	
C25	0.5235 (4)	0.0111 (5)	0.9773 (5)	0.104 (3)	
H25	0.539693	−0.055026	0.961602	0.124*	
C26	0.5314 (4)	0.6410 (6)	0.8935 (4)	0.0804 (18)	
C27	0.4446 (3)	0.6360 (4)	0.8759 (3)	0.0664 (14)	
H27	0.428202	0.641918	0.823245	0.080*	
C28	0.4105 (4)	0.7320 (5)	0.9101 (3)	0.0778 (16)	
H28A	0.424075	0.724288	0.962241	0.093*	
H28B	0.434021	0.798545	0.897435	0.093*	
C29	0.3254 (5)	0.7458 (6)	0.8891 (5)	0.098 (2)	
H29	0.301566	0.675093	0.894081	0.117*	
C30	0.3004 (6)	0.7846 (11)	0.8102 (6)	0.195 (5)	
H30A	0.317410	0.733412	0.778404	0.293*	
H30B	0.246106	0.789565	0.798264	0.293*	
H30C	0.321961	0.854958	0.804816	0.293*	
C31	0.2988 (6)	0.8255 (8)	0.9385 (7)	0.164 (4)	
H31A	0.244850	0.833614	0.924712	0.246*	
H31B	0.311856	0.799333	0.987370	0.246*	
H31C	0.322687	0.894820	0.935077	0.246*	
C32	0.3922 (3)	0.4556 (4)	0.8372 (3)	0.0645 (13)	
H32A	0.435248	0.441562	0.814871	0.077*	
H32B	0.353162	0.490151	0.801309	0.077*	
C33	0.3628 (3)	0.3497 (4)	0.8590 (3)	0.0593 (12)	
C34	0.3872 (3)	0.2514 (5)	0.8345 (3)	0.0728 (15)	
H34	0.423115	0.252114	0.805316	0.087*	
C35	0.3586 (4)	0.1516 (5)	0.8532 (4)	0.0872 (19)	
H35	0.374557	0.086691	0.835592	0.105*	
C36	0.3074 (4)	0.1500 (5)	0.8971 (4)	0.0847 (19)	
H36	0.288831	0.083470	0.909771	0.102*	
C37	0.2824 (3)	0.2463 (5)	0.9235 (4)	0.0759 (16)	
H37	0.249014	0.244196	0.955244	0.091*	
C38	0.3078 (3)	0.3458 (4)	0.9020 (3)	0.0606 (13)	
N3	0.5503 (2)	0.3089 (4)	0.9548 (2)	0.0644 (11)	
N4	0.4156 (2)	0.5315 (3)	0.8985 (2)	0.0529 (9)	
H4	0.369103	0.549857	0.915387	0.063*	
O4	0.5687 (2)	0.5731 (4)	0.9349 (2)	0.0770 (11)	
O5	0.5606 (4)	0.7171 (7)	0.8649 (4)	0.164 (4)	
O6	0.2817 (2)	0.4428 (3)	0.9223 (2)	0.0803 (11)	
H6A	0.250728	0.431603	0.947898	0.120*	

### Atomic displacement parameters (Å^2^)

**Table:**

	*U*^11^	*U*^22^	*U*^33^	*U*^12^	*U*^13^	*U*^23^
Cd1	0.0543 (4)	0.0500 (4)	0.0664 (4)	0.000	0.0272 (3)	0.000
C1	0.072 (4)	0.118 (6)	0.096 (5)	−0.017 (4)	0.024 (4)	−0.012 (4)
C2	0.101 (7)	0.160 (10)	0.143 (8)	−0.066 (7)	0.015 (6)	−0.024 (8)
C3	0.197 (12)	0.117 (8)	0.126 (8)	−0.089 (9)	0.064 (8)	−0.035 (7)
C4	0.171 (8)	0.073 (5)	0.082 (5)	−0.042 (5)	0.048 (5)	−0.010 (4)
C5	0.105 (4)	0.051 (3)	0.058 (3)	−0.009 (3)	0.031 (3)	−0.002 (2)
C6	0.303 (19)	0.057 (4)	0.105 (9)	−0.026 (6)	0.101 (11)	−0.008 (5)
C7	0.072 (4)	0.066 (3)	0.085 (4)	0.019 (3)	0.043 (3)	0.027 (3)
C8	0.085 (4)	0.051 (4)	0.078 (5)	0.007 (3)	0.044 (4)	0.012 (3)
C9	0.121 (5)	0.056 (4)	0.080 (4)	0.017 (3)	0.047 (4)	0.009 (3)
C10	0.184 (8)	0.091 (5)	0.095 (5)	−0.019 (6)	0.045 (5)	−0.015 (5)
C11	0.220 (11)	0.090 (6)	0.136 (8)	−0.023 (7)	0.012 (7)	−0.021 (6)
C12	0.244 (13)	0.261 (15)	0.145 (9)	−0.056 (11)	0.097 (10)	−0.084 (9)
C13	0.093 (4)	0.070 (4)	0.103 (5)	0.014 (3)	0.056 (4)	0.029 (3)
C14	0.074 (4)	0.067 (4)	0.057 (3)	0.012 (3)	0.029 (3)	0.015 (3)
C15	0.083 (4)	0.080 (4)	0.062 (3)	−0.012 (4)	0.016 (3)	0.015 (4)
C16	0.141 (7)	0.063 (4)	0.069 (4)	−0.006 (4)	0.036 (4)	0.008 (3)
C17	0.144 (7)	0.075 (5)	0.077 (5)	0.031 (5)	0.037 (5)	0.021 (4)
C18	0.106 (5)	0.107 (6)	0.079 (4)	0.010 (5)	0.002 (4)	0.030 (4)
C19	0.117 (5)	0.062 (4)	0.054 (3)	−0.008 (4)	0.006 (3)	0.013 (3)
N1	0.072 (3)	0.070 (3)	0.064 (3)	−0.019 (2)	0.022 (2)	−0.003 (2)
N2	0.067 (3)	0.052 (2)	0.061 (2)	0.007 (2)	0.033 (2)	0.011 (2)
O1	0.073 (2)	0.078 (3)	0.074 (2)	0.0225 (19)	0.029 (2)	0.006 (2)
O2	0.099 (3)	0.117 (3)	0.106 (3)	0.057 (3)	0.052 (3)	0.039 (3)
O3	0.182 (5)	0.094 (4)	0.090 (3)	−0.043 (3)	−0.027 (3)	0.017 (3)
Cd2	0.0580 (4)	0.0627 (4)	0.0563 (4)	0.000	0.0145 (3)	0.000
C20	0.075 (4)	0.082 (5)	0.071 (4)	0.008 (4)	0.011 (3)	−0.012 (4)
C21	0.086 (4)	0.112 (6)	0.088 (5)	0.018 (4)	0.025 (3)	−0.026 (4)
C22	0.089 (5)	0.088 (6)	0.109 (6)	0.022 (4)	−0.002 (4)	−0.034 (4)
C23	0.064 (4)	0.081 (5)	0.092 (5)	0.015 (3)	−0.013 (3)	−0.018 (4)
C24	0.053 (3)	0.064 (4)	0.073 (4)	0.003 (2)	−0.007 (2)	−0.006 (3)
C25	0.099 (7)	0.054 (4)	0.141 (8)	0.006 (3)	−0.018 (4)	−0.011 (4)
C26	0.085 (5)	0.091 (5)	0.070 (4)	−0.035 (4)	0.028 (4)	−0.007 (4)
C27	0.086 (4)	0.066 (3)	0.046 (3)	−0.020 (3)	0.011 (3)	0.008 (2)
C28	0.104 (5)	0.055 (3)	0.068 (4)	−0.013 (3)	0.001 (3)	0.009 (3)
C29	0.110 (6)	0.056 (4)	0.119 (6)	0.007 (4)	0.005 (5)	0.005 (4)
C30	0.150 (9)	0.275 (15)	0.136 (9)	0.038 (9)	−0.033 (7)	0.031 (9)
C31	0.180 (10)	0.124 (8)	0.189 (11)	0.073 (7)	0.038 (8)	0.017 (7)
C32	0.071 (3)	0.068 (3)	0.053 (3)	−0.010 (3)	0.009 (2)	−0.003 (3)
C33	0.055 (3)	0.054 (3)	0.065 (3)	−0.011 (2)	0.001 (2)	−0.009 (2)
C34	0.069 (3)	0.070 (4)	0.076 (4)	0.002 (3)	0.006 (3)	−0.016 (3)
C35	0.088 (5)	0.057 (4)	0.113 (5)	−0.004 (3)	0.010 (4)	−0.018 (3)
C36	0.067 (4)	0.055 (4)	0.126 (6)	−0.008 (3)	0.006 (4)	0.005 (4)
C37	0.054 (3)	0.072 (4)	0.101 (5)	−0.011 (3)	0.014 (3)	0.009 (4)
C38	0.058 (3)	0.053 (3)	0.070 (3)	−0.011 (2)	0.011 (3)	−0.009 (3)
N3	0.055 (3)	0.072 (3)	0.064 (3)	−0.001 (2)	0.008 (2)	−0.009 (2)
N4	0.058 (2)	0.052 (2)	0.050 (2)	−0.0103 (18)	0.0133 (18)	−0.0006 (18)
O4	0.061 (2)	0.091 (3)	0.083 (3)	−0.013 (2)	0.026 (2)	−0.005 (2)
O5	0.157 (5)	0.182 (8)	0.148 (5)	−0.098 (6)	0.021 (4)	0.078 (6)
O6	0.071 (2)	0.059 (2)	0.124 (3)	−0.0123 (18)	0.049 (2)	−0.011 (2)

### Geometric parameters (Å, º)

**Table:**

Cd1—N2^i^	2.315 (4)	Cd2—O4^ii^	2.322 (4)
Cd1—N2	2.315 (4)	Cd2—O4	2.322 (4)
Cd1—N1^i^	2.341 (4)	Cd2—N4^ii^	2.339 (4)
Cd1—N1	2.341 (4)	Cd2—N4	2.339 (4)
Cd1—O1^i^	2.346 (3)	Cd2—N3	2.351 (5)
Cd1—O1	2.346 (3)	Cd2—N3^ii^	2.352 (5)
C1—N1	1.320 (8)	C20—N3	1.321 (8)
C1—C2	1.400 (12)	C20—C21	1.420 (10)
C1—H1	0.9300	C20—H20	0.9300
C2—C3	1.324 (15)	C21—C22	1.353 (10)
C2—H2	0.9300	C21—H21	0.9300
C3—C4	1.393 (15)	C22—C23	1.388 (10)
C3—H3	0.9300	C22—H22	0.9300
C4—C5	1.393 (9)	C23—C24	1.408 (8)
C4—C6	1.408 (12)	C23—C25	1.459 (9)
C5—N1	1.347 (7)	C24—N3	1.333 (7)
C5—C5^i^	1.449 (12)	C24—C24^ii^	1.458 (12)
C6—C6^i^	1.30 (2)	C25—C25^ii^	1.318 (16)
C6—H6	0.9300	C25—H25	0.9300
C7—O1	1.251 (6)	C26—O4	1.242 (8)
C7—O2	1.252 (6)	C26—O5	1.246 (8)
C7—C8	1.529 (9)	C26—C27	1.533 (8)
C8—C9	1.503 (10)	C27—N4	1.477 (6)
C8—N2	1.506 (8)	C27—C28	1.529 (8)
C8—H8	0.9800	C27—H27	0.9800
C9—C10	1.544 (10)	C28—C29	1.514 (10)
C9—H9A	0.9700	C28—H28A	0.9700
C9—H9B	0.9700	C28—H28B	0.9700
C10—C12	1.482 (12)	C29—C31	1.494 (13)
C10—C11	1.543 (11)	C29—C30	1.540 (13)
C10—H10	0.9800	C29—H29	0.9800
C11—H11A	0.9600	C30—H30A	0.9600
C11—H11B	0.9600	C30—H30B	0.9600
C11—H11C	0.9600	C30—H30C	0.9600
C12—H12A	0.9600	C31—H31A	0.9600
C12—H12B	0.9600	C31—H31B	0.9600
C12—H12C	0.9600	C31—H31C	0.9600
C13—N2	1.489 (6)	C32—N4	1.477 (6)
C13—C14	1.504 (7)	C32—C33	1.491 (7)
C13—H13A	0.9700	C32—H32A	0.9700
C13—H13B	0.9700	C32—H32B	0.9700
C14—C15	1.355 (10)	C33—C34	1.393 (7)
C14—C19	1.380 (8)	C33—C38	1.402 (7)
C15—C16	1.360 (10)	C34—C35	1.400 (8)
C15—H15	0.9300	C34—H34	0.9300
C16—C17	1.354 (9)	C35—C36	1.358 (10)
C16—H16	0.9300	C35—H35	0.9300
C17—C18	1.356 (10)	C36—C37	1.391 (9)
C17—H17	0.9300	C36—H36	0.9300
C18—C19	1.369 (9)	C37—C38	1.391 (8)
C18—H18	0.9300	C37—H37	0.9300
C19—O3	1.359 (7)	C38—O6	1.360 (6)
N2—H2A	0.9800	N4—H4	0.9800
O3—H3A	0.8200	O6—H6A	0.8200
			
N2^i^—Cd1—N2	142.70 (19)	O4^ii^—Cd2—O4	110.6 (2)
N2^i^—Cd1—N1^i^	102.22 (15)	O4^ii^—Cd2—N4^ii^	72.40 (14)
N2—Cd1—N1^i^	107.96 (14)	O4—Cd2—N4^ii^	84.68 (14)
N2^i^—Cd1—N1	107.96 (14)	O4^ii^—Cd2—N4	84.68 (14)
N2—Cd1—N1	102.22 (15)	O4—Cd2—N4	72.40 (14)
N1^i^—Cd1—N1	71.2 (2)	N4^ii^—Cd2—N4	139.38 (18)
N2^i^—Cd1—O1^i^	73.01 (13)	O4^ii^—Cd2—N3	160.06 (16)
N2—Cd1—O1^i^	85.98 (14)	O4—Cd2—N3	89.30 (16)
N1^i^—Cd1—O1^i^	88.94 (16)	N4^ii^—Cd2—N3	110.19 (13)
N1—Cd1—O1^i^	159.98 (16)	N4—Cd2—N3	102.76 (14)
N2^i^—Cd1—O1	85.98 (14)	O4^ii^—Cd2—N3^ii^	89.30 (17)
N2—Cd1—O1	73.01 (13)	O4—Cd2—N3^ii^	160.06 (16)
N1^i^—Cd1—O1	159.98 (16)	N4^ii^—Cd2—N3^ii^	102.76 (14)
N1—Cd1—O1	88.94 (16)	N4—Cd2—N3^ii^	110.18 (13)
O1^i^—Cd1—O1	111.0 (2)	N3—Cd2—N3^ii^	70.8 (2)
N1—C1—C2	121.5 (8)	N3—C20—C21	120.3 (8)
N1—C1—H1	119.2	N3—C20—H20	119.8
C2—C1—H1	119.2	C21—C20—H20	119.8
C3—C2—C1	118.9 (10)	C22—C21—C20	119.9 (7)
C3—C2—H2	120.6	C22—C21—H21	120.0
C1—C2—H2	120.6	C20—C21—H21	120.0
C2—C3—C4	122.5 (9)	C21—C22—C23	119.8 (6)
C2—C3—H3	118.7	C21—C22—H22	120.1
C4—C3—H3	118.7	C23—C22—H22	120.1
C3—C4—C5	114.9 (8)	C22—C23—C24	117.3 (7)
C3—C4—C6	124.6 (9)	C22—C23—C25	123.2 (7)
C5—C4—C6	120.5 (9)	C24—C23—C25	119.6 (7)
N1—C5—C4	123.6 (7)	N3—C24—C23	122.5 (6)
N1—C5—C5^i^	118.3 (3)	N3—C24—C24^ii^	118.3 (3)
C4—C5—C5^i^	118.1 (5)	C23—C24—C24^ii^	119.2 (4)
C6^i^—C6—C4	121.3 (6)	C25^ii^—C25—C23	121.2 (4)
C6^i^—C6—H6	119.3	C25^ii^—C25—H25	119.4
C4—C6—H6	119.3	C23—C25—H25	119.4
O1—C7—O2	123.6 (6)	O4—C26—O5	123.5 (7)
O1—C7—C8	120.5 (5)	O4—C26—C27	120.6 (5)
O2—C7—C8	115.9 (6)	O5—C26—C27	115.9 (7)
C9—C8—N2	110.3 (5)	N4—C27—C28	110.5 (4)
C9—C8—C7	109.8 (5)	N4—C27—C26	112.2 (5)
N2—C8—C7	110.9 (5)	C28—C27—C26	110.8 (5)
C9—C8—H8	108.6	N4—C27—H27	107.7
N2—C8—H8	108.6	C28—C27—H27	107.7
C7—C8—H8	108.6	C26—C27—H27	107.7
C8—C9—C10	116.6 (6)	C29—C28—C27	116.6 (5)
C8—C9—H9A	108.1	C29—C28—H28A	108.2
C10—C9—H9A	108.1	C27—C28—H28A	108.2
C8—C9—H9B	108.1	C29—C28—H28B	108.2
C10—C9—H9B	108.1	C27—C28—H28B	108.2
H9A—C9—H9B	107.3	H28A—C28—H28B	107.3
C12—C10—C11	110.7 (8)	C31—C29—C28	110.2 (7)
C12—C10—C9	113.2 (9)	C31—C29—C30	109.4 (8)
C11—C10—C9	110.7 (7)	C28—C29—C30	111.8 (8)
C12—C10—H10	107.3	C31—C29—H29	108.5
C11—C10—H10	107.3	C28—C29—H29	108.5
C9—C10—H10	107.3	C30—C29—H29	108.5
C10—C11—H11A	109.5	C29—C30—H30A	109.5
C10—C11—H11B	109.5	C29—C30—H30B	109.5
H11A—C11—H11B	109.5	H30A—C30—H30B	109.5
C10—C11—H11C	109.5	C29—C30—H30C	109.5
H11A—C11—H11C	109.5	H30A—C30—H30C	109.5
H11B—C11—H11C	109.5	H30B—C30—H30C	109.5
C10—C12—H12A	109.5	C29—C31—H31A	109.5
C10—C12—H12B	109.5	C29—C31—H31B	109.5
H12A—C12—H12B	109.5	H31A—C31—H31B	109.5
C10—C12—H12C	109.5	C29—C31—H31C	109.5
H12A—C12—H12C	109.5	H31A—C31—H31C	109.5
H12B—C12—H12C	109.5	H31B—C31—H31C	109.5
N2—C13—C14	113.7 (4)	N4—C32—C33	113.2 (4)
N2—C13—H13A	108.8	N4—C32—H32A	108.9
C14—C13—H13A	108.8	C33—C32—H32A	108.9
N2—C13—H13B	108.8	N4—C32—H32B	108.9
C14—C13—H13B	108.8	C33—C32—H32B	108.9
H13A—C13—H13B	107.7	H32A—C32—H32B	107.7
C15—C14—C19	117.1 (5)	C34—C33—C38	118.0 (5)
C15—C14—C13	120.4 (6)	C34—C33—C32	120.5 (5)
C19—C14—C13	122.4 (6)	C38—C33—C32	121.4 (5)
C14—C15—C16	123.0 (6)	C33—C34—C35	121.0 (6)
C14—C15—H15	118.5	C33—C34—H34	119.5
C16—C15—H15	118.5	C35—C34—H34	119.5
C17—C16—C15	118.9 (7)	C36—C35—C34	119.8 (6)
C17—C16—H16	120.6	C36—C35—H35	120.1
C15—C16—H16	120.6	C34—C35—H35	120.1
C16—C17—C18	120.4 (6)	C35—C36—C37	120.9 (6)
C16—C17—H17	119.8	C35—C36—H36	119.5
C18—C17—H17	119.8	C37—C36—H36	119.5
C17—C18—C19	120.0 (6)	C36—C37—C38	119.4 (6)
C17—C18—H18	120.0	C36—C37—H37	120.3
C19—C18—H18	120.0	C38—C37—H37	120.3
O3—C19—C18	119.8 (6)	O6—C38—C37	122.1 (5)
O3—C19—C14	119.4 (6)	O6—C38—C33	117.2 (5)
C18—C19—C14	120.7 (6)	C37—C38—C33	120.7 (5)
C1—N1—C5	118.6 (5)	C20—N3—C24	120.1 (6)
C1—N1—Cd1	125.3 (5)	C20—N3—Cd2	123.6 (5)
C5—N1—Cd1	116.1 (4)	C24—N3—Cd2	116.3 (4)
C13—N2—C8	110.9 (4)	C32—N4—C27	112.5 (4)
C13—N2—Cd1	115.4 (3)	C32—N4—Cd2	117.3 (3)
C8—N2—Cd1	109.6 (3)	C27—N4—Cd2	109.1 (3)
C13—N2—H2A	106.8	C32—N4—H4	105.7
C8—N2—H2A	106.8	C27—N4—H4	105.7
Cd1—N2—H2A	106.8	Cd2—N4—H4	105.7
C7—O1—Cd1	116.1 (4)	C26—O4—Cd2	115.8 (4)
C19—O3—H3A	109.5	C38—O6—H6A	109.5
			
N1—C1—C2—C3	−0.4 (15)	N3—C20—C21—C22	1.6 (10)
C1—C2—C3—C4	0.2 (17)	C20—C21—C22—C23	−2.7 (10)
C2—C3—C4—C5	−0.4 (15)	C21—C22—C23—C24	2.3 (9)
C2—C3—C4—C6	−179.3 (11)	C21—C22—C23—C25	−177.3 (7)
C3—C4—C5—N1	0.7 (10)	C22—C23—C24—N3	−0.8 (8)
C6—C4—C5—N1	179.6 (8)	C25—C23—C24—N3	178.7 (6)
C3—C4—C5—C5^i^	179.1 (7)	C22—C23—C24—C24^ii^	178.9 (6)
C6—C4—C5—C5^i^	−2.0 (11)	C25—C23—C24—C24^ii^	−1.5 (9)
C3—C4—C6—C6^i^	177.1 (14)	C22—C23—C25—C25^ii^	180.0 (9)
C5—C4—C6—C6^i^	−2 (2)	C24—C23—C25—C25^ii^	0.4 (13)
O1—C7—C8—C9	96.8 (7)	O4—C26—C27—N4	−13.5 (8)
O2—C7—C8—C9	−80.3 (7)	O5—C26—C27—N4	167.5 (6)
O1—C7—C8—N2	−25.4 (8)	O4—C26—C27—C28	110.5 (6)
O2—C7—C8—N2	157.5 (5)	O5—C26—C27—C28	−68.5 (8)
N2—C8—C9—C10	−64.3 (8)	N4—C27—C28—C29	−62.8 (6)
C7—C8—C9—C10	173.2 (6)	C26—C27—C28—C29	172.3 (5)
C8—C9—C10—C12	−63.4 (10)	C27—C28—C29—C31	167.2 (7)
C8—C9—C10—C11	171.6 (7)	C27—C28—C29—C30	−71.0 (9)
N2—C13—C14—C15	−135.5 (6)	N4—C32—C33—C34	−133.4 (5)
N2—C13—C14—C19	48.4 (8)	N4—C32—C33—C38	49.2 (6)
C19—C14—C15—C16	−0.7 (9)	C38—C33—C34—C35	−1.0 (8)
C13—C14—C15—C16	−177.0 (6)	C32—C33—C34—C35	−178.4 (5)
C14—C15—C16—C17	0.8 (10)	C33—C34—C35—C36	−1.4 (9)
C15—C16—C17—C18	−1.4 (11)	C34—C35—C36—C37	0.5 (10)
C16—C17—C18—C19	1.8 (11)	C35—C36—C37—C38	2.8 (10)
C17—C18—C19—O3	−179.0 (7)	C36—C37—C38—O6	175.8 (5)
C17—C18—C19—C14	−1.7 (10)	C36—C37—C38—C33	−5.3 (8)
C15—C14—C19—O3	178.5 (5)	C34—C33—C38—O6	−176.6 (4)
C13—C14—C19—O3	−5.3 (9)	C32—C33—C38—O6	0.7 (7)
C15—C14—C19—C18	1.1 (9)	C34—C33—C38—C37	4.4 (8)
C13—C14—C19—C18	177.4 (5)	C32—C33—C38—C37	−178.3 (5)
C2—C1—N1—C5	0.7 (10)	C21—C20—N3—C24	−0.2 (9)
C2—C1—N1—Cd1	−178.0 (6)	C21—C20—N3—Cd2	−178.8 (5)
C4—C5—N1—C1	−0.9 (8)	C23—C24—N3—C20	−0.2 (8)
C5^i^—C5—N1—C1	−179.3 (6)	C24^ii^—C24—N3—C20	−179.9 (6)
C4—C5—N1—Cd1	177.9 (5)	C23—C24—N3—Cd2	178.6 (4)
C5^i^—C5—N1—Cd1	−0.5 (7)	C24^ii^—C24—N3—Cd2	−1.2 (7)
C14—C13—N2—C8	−176.2 (5)	C33—C32—N4—C27	179.9 (4)
C14—C13—N2—Cd1	58.4 (6)	C33—C32—N4—Cd2	52.3 (5)
C9—C8—N2—C13	143.7 (5)	C28—C27—N4—C32	134.3 (5)
C7—C8—N2—C13	−94.4 (6)	C26—C27—N4—C32	−101.4 (5)
C9—C8—N2—Cd1	−87.7 (5)	C28—C27—N4—Cd2	−93.8 (4)
C7—C8—N2—Cd1	34.2 (6)	C26—C27—N4—Cd2	30.4 (5)
O2—C7—O1—Cd1	179.1 (5)	O5—C26—O4—Cd2	166.8 (6)
C8—C7—O1—Cd1	2.2 (7)	C27—C26—O4—Cd2	−12.1 (8)

Symmetry codes: (i) −*x*+1, *y*, −*z*+1; (ii) −*x*+1, *y*, −*z*+2.

### Hydrogen-bond geometry (Å, º)

**Table:**

*D*—H···*A*	*D*—H	H···*A*	*D*···*A*	*D*—H···*A*
O6—H6*A*···O2^iii^	0.82	1.83	2.645 (5)	174
N4—H4···O6	0.98	2.07	2.763 (5)	126
N2—H2*A*···O3	0.98	2.09	2.795 (6)	127
O3—H3*A*···O5	0.82	2.33	2.951 (9)	133
C28—H28*A*···O4^ii^	0.97	2.67	3.470 (7)	141

Symmetry codes: (ii) −*x*+1, *y*, −*z*+2; (iii) −*x*+1/2, *y*−1/2, −*z*+3/2.
